# Exploring the Molecular Tumor Microenvironment and Translational Biomarkers in Brain Metastases of Non-Small-Cell Lung Cancer

**DOI:** 10.3390/ijms25042044

**Published:** 2024-02-07

**Authors:** Jiexi Wen, Jie-Zeng Yu, Catherine Liu, A. Aziz O. Ould Ismail, Weijie Ma

**Affiliations:** 1 Department of Pathology and Laboratory Medicine, Dartmouth-Hitchcock Medical Center, Geisel School of Medicine at Dartmouth, Lebanon, NH 03756, USA; 2 Division of Hematology/Oncology, Department of Medicine, University of California at San Francisco, San Francisco, CA 94143, USA; 3 School of Medicine and Dentistry, University of Rochester, Rochester, NY 14642, USA

**Keywords:** brain metastases, non-small-cell lung cancer, biomarker, tumor microenvironment, molecular cancer

## Abstract

Brain metastases represent a significant clinical challenge in the treatment of non-small-cell lung cancer (NSCLC), often leading to a severe decline in patient prognosis and survival. Recent advances in imaging and systemic treatments have increased the detection rates of brain metastases, yet clinical outcomes remain dismal due to the complexity of the metastatic tumor microenvironment (TME) and the lack of specific biomarkers for early detection and targeted therapy. The intricate interplay between NSCLC tumor cells and the surrounding TME in brain metastases is pivotal, influencing tumor progression, immune evasion, and response to therapy. This underscores the necessity for a deeper understanding of the molecular underpinnings of brain metastases, tumor microenvironment, and the identification of actionable biomarkers that can inform multimodal treatment approaches. The goal of this review is to synthesize current insights into the TME and elucidate molecular mechanisms in NSCLC brain metastases. Furthermore, we will explore the promising horizon of emerging biomarkers, both tissue- and liquid-based, that hold the potential to radically transform the treatment strategies and the enhancement of patient outcomes.

## 1. Introduction

Non-small-cell lung cancer (NSCLC), the predominant form of lung cancer, continues to be a profound health concern across the globe and a leading cause of morbidity and mortality in the United States [1]. This concern is magnified by NSCLC’s tendency to metastasize, most notably to the brain, an attribute that significantly influences its mortality statistics. In NSCLC patients, the development of brain metastases is witnessed in around 40% cases, making up nearly half of all brain metastases diagnoses and indicating a particularly severe prognosis [2]. Brain metastases often appear as multiple lesions, although one-third of these patients present with single lesions [3]. Untreated, the median survival for those with brain metastases from NSCLC ranges from just 1 to 2 months, underscoring the urgency and complexity of this clinical problem [4].

The key prognostic factors in NSCLC include the patient’s age, extracranial tumor activity, number and nature of brain metastases, and molecular subtype of the primary tumor [5]. The emergence of brain metastases consistently signals a bleak prognosis, with median survival times averaging between 3 and 6 months [6]. The tumor microenvironment (TME) is a key factor in driving the complex pathophysiology of NSCLC, starting at the primary site to involving lymphovascular channels to arriving and culminating in the brain [7]. The TME, a multifaceted network comprising both cellular and non-cellular constituents such as blood vessels, fibroblasts, immune cells, bone marrow-derived inflammatory cells, signaling molecules, and the extracellular matrix (ECM), facilitates a symbiotic relationship with tumor cells [8,9]. This interaction modulates tumor progression by endorsing angiogenesis and impeding immune response. Remarkably, the brain’s exclusive microenvironment retains its distinct characteristics following brain metastases, differentiating it from other organs [10]. Furthermore, the unique structure of the blood–brain barrier (BBB) restricts most chemotherapeutic agents from reaching intracranial lesions, limiting their therapeutic application [11]. Traditional treatment for brain metastases from NSCLC, such as surgical resection, is often constrained by risks associated with lesion numbers and locations in the brain. Other approaches such as whole-brain and stereotactic radiotherapy, despite their efficacy, have potential adverse effects like radionecrosis, leading to neurotoxicity [12]. In this comprehensive review, we explore the current state of knowledge and understanding of the TME’s role in NSCLC brain metastasis, focusing on underlying molecular mechanisms and predictive biomarkers. Moreover, we investigate the promising potential of these biomarkers in paving the way for innovative diagnostic and therapeutic strategies, offering hope for a more nuanced and targeted approach to NSCLC.

## 2. Tumor Microenvironment in Brain Metastases of NSCLC

### 2.1. Overview of TME in Brain Metastases

**Figure 1:**
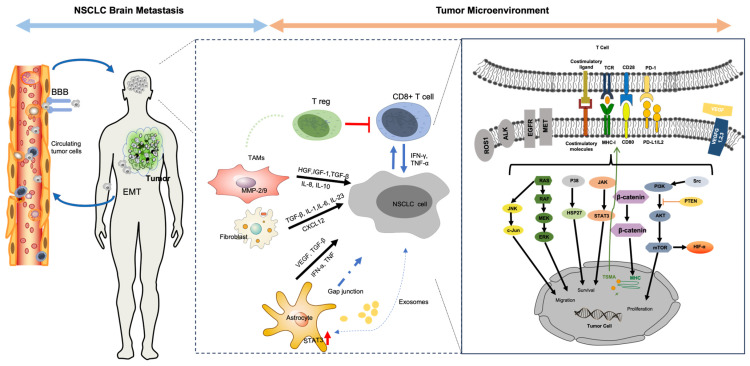
Schema of interactions between TME and NSCLC brain metastases. Abbreviations: ALK: anaplastic lymphoma kinase; AKT: protein kinase B; BBB: blood–brain barrier; EGFR: epidermal growth factor receptor; EMT: epithelial-mesenchymal transition; HGF: hepatocyte growth factor; HIF-α: hypoxia-inducible factor-alpha; HSP27: heat shock protein 27; JAK: Janus kinase; JNK: c-Jun N-terminal kinase; TGF-β: transforming growth factor-beta; IFN-α: interferon-alpha; IL: interleukin; PI3K: phosphoinositide 3-kinase; PTEN: phosphatase and tensin homolog; NSCLC: non-small-cell lung cancer; STAT3: signal transducer and activator of transcription 3; TAMs: tumor-associated macrophages; TCR: T cell receptor; TNF: tumor necrosis factor; MAPK: mitogen-activated protein kinase; MMP: matrix metalloproteinase; MET: mesenchymal–epithelial transition factor; ROS1: c-ros oncogene 1, receptor tyrosine kinase; VEGF: vascular endothelial growth factor.

Brain metastases in NSCLC represent a complex and dynamic interplay between tumor cells, immune cells, and the unique brain TME. Metastasis is not an inherent trait of a specific tumor but rather multi-step and multidimensional processes influenced by factors such as mutations, epigenetic changes, and the presence of growth factors [3,13]. As illustrated in Figure 1, starting from the invasion of the primary tumor’s basement membrane, the metastatic process involves epithelial-to-mesenchymal transition (EMT) and intravasation into local blood and lymphatic vessels, eventually leading to circulating tumor cells (CTCs) surviving defensive mechanisms in the circulatory system. CTCs then face the considerable challenge of crossing the blood–brain barrier (BBB), a tightly regulated structure. Many brain metastases show BBB deterioration, suggesting an active role of CTCs in compromising these junctions. The mechanism of BBB crossing mimics aspects of leukocyte extravasation and involves adhesion and interactions with endothelial markers such as CD15 and CD15s [14]. These CTCs further release factors, such as CXCL12/CXCR4, MMP-1, and VEGF, that reciprocally degrade the BBB and facilitate their invasion [15]. Within the TME of NSCLC, hypoxia can induce HIF-1a, which elevates midkine (MDK) levels, thus promoting cell proliferation and further inducing EMT through Notch signaling. Other molecular players such as zinc transporter 4 also regulate EMT via the Snail–N-cadherin signaling axis, offering potential avenues for therapeutic interventions [16]. Upon invading the brain, tumor cells must alter the brain TME in a fashion that promotes tumor invasion and growth. To do this, tumor cells phenotypically modify local cells. These modified local cells can then go on to further stimulate tumor cells and the TME in a reciprocal fashion [17]. The brain TME is enriched with various local brain cells such as microglia and astrocytes that have been shown to demonstrate this reciprocal interaction. Initially, microglia adopt an anti-tumor M1 phenotype but gradually transition to a pro-tumor M2 phenotype under the influence of tumor-derived cytokines, specifically STAT3 [18]. This M1-to-M2 shift has substantial implications, as a higher M2:M1 ratio is strongly associated with brain metastases. Astrocytes also transform, evolving from an initial anti-tumor role to a pro-tumor one [19]. This transition is mediated by the formation of gap junctions between tumor cells and astrocytes which facilitate the transfer of messenger molecules that induce pro-tumor phenotypes in astrocytes. Additionally, the brain TME is further characterized by reduced T cell infiltration and changed diversity in T cell receptor (TCR) sequences compared to primary tumors, highlighting a state of immunosuppression [20,21]. Other factors that remain poorly understood include the influence of cancer-associated fibroblasts (CAFs) and vascular interactions, specifically vascular co-option, which are thought to be integral to tumor growth within the brain. Vascular co-option, the process where tumor cells physically adhere to pre-existing vessels, often precedes angiogenesis and is critical for tumor establishment and growth. Understanding these complex interactions between tumor cells and the TME in brain metastases from NSCLC holds both significant prognostic and therapeutic implications as it could pave the way for more targeted and effective therapeutic strategies in managing NSCLC brain metastases.

### 2.2. Cellular Components

The TME of brain metastases originating from NSCLC exhibits distinct characteristics divergent from those of the primary NSCLC site. This TME is composed of a constellation of cellular elements, including not only tumor cells but also a milieu of stromal components such as fibroblasts, astrocytes, and an array of immune cells, including microglia, macrophages, and lymphocytes [22]. Concurrently, non-cellular elements, predominantly the extracellular matrix (ECM), significantly contribute to the architecture of this environment [23]. The brain’s microenvironment is subject to transformative changes following tumor colonization, which implicates both the ECM and the resident tissue cells. Given the brain microenvironment’s critical role in shaping the trajectory of brain metastases, it becomes imperative to delineate the interactions between metastatic tumor cells and the diverse constituents of the brain’s ecological niche.

In general, brain metastases are characterized by an immunosuppressive tumor microenvironment (TME). This is evidenced by the suppression of immune-related signaling pathways, diminished expression of immune checkpoints, reduced infiltration by CD8+ T cells and cytotoxic lymphocytes, and a higher proportion of immunosuppressive M2 macrophages [3,10,24,25]. Kudo et al. [26] conducted a thorough immune profiling and sequencing analysis of NSCLC, focusing on both primary tumors and their paired brain metastases. Their findings revealed a predominant sharing of somatic hot spot mutations across these tumor sites. Notably, they observed a more pronounced inhibition of certain signaling pathways in brain metastases, namely those related to dendritic cell maturation, Th1 response, and leukocyte extravasation, compared to primary tumors. Moreover, the proinflammatory cell adhesion molecule, vascular cell adhesion protein 1, was markedly downregulated in brain metastases relative to primary tumors. The study also highlighted a distinct immune landscape in brain metastases, characterized by a reduced T cell presence and increased macrophage infiltration, in contrast to primary tumors. Furthermore, an expansion of T cell clones was observed in 64% of brain metastases when compared to their corresponding primary tumors. A notable decrease in the interferon-γ-related gene signature and an upregulation of anti-inflammatory markers such as TOLLIP and HLA g in brain metastases indicate a shift in the inflammatory profile of these tumors [27]. Furthermore, while TCR repertoires were largely shared between paired brain metastases and primary tumors, T cell densities were sparse in the metastases. Zhang et al. [28] provided further insight into the brain TME, highlighting an extensive remodeling process that fosters an immunosuppressive and fibrogenic niche conducive to brain metastasis establishment. This altered brain TME is typified by attenuated antigen presentation, compromised B/T cell functionality, an uptick in neutrophils and M2-type macrophages, along with immature microglia and reactive astrocytes. They pinpoint fibrosis as a hallmark of the brain tumor microenvironment. Differential gene expression and network analyses have illuminated that fibrosis and immune regulation represent the major disrupted functional modules within both lung and brain TMEs. Recent studies applying single-cell transcriptomic sequencing were able to unlock novel key molecular features of individual metastatic cells in brain metastases [29]. The researchers delineated pronounced disparities in the immune landscapes of primary and metastatic brain tumors, uncovering a distinctive gene signature linked to metabolic functions and cell adhesion in the metastases. Notably, there was a marked increase in the prevalence and concentration of CD16+ NK cells, neutrophils, macrophages, classical monocytes, and T cells in brain metastases compared to glioblastoma, coupled with a reduction in dendritic cells and non-classical monocytes. The study also revealed greater heterogeneity among the immune cells of different patients than within the cells from individual patients.

#### 2.2.1. T Cells

Despite the distinct genetic and immunological landscape of brain metastases, the composition and function of T cells, particularly regulatory T cells (Tregs), are largely unexplored. In NSCLC-associated brain metastases, Tregs contribute to an immunosuppressive tumor microenvironment, often appearing in increased numbers alongside elevated levels of myeloid-derived suppressor cells (MDSCs), monocytes PD-L1 and IL-6 in the peripheral blood of affected patients [30,31]. Interestingly, Wainwright et al. [32] identified Helios+ thymus-derived natural Tregs as the predominant subset infiltrating brain tumors, further accumulating in the cervical lymph nodes through meningeal lymphatic drainage from the CNS. Notably, a high ratio of infiltrating Tregs to CD8+ T cells in tumor tissues is correlated with poor prognoses [33]. In contrast, brain metastases resulting from NSCLC show a reduced abundance of CD8+ T cells, CD4+ T cells, and Tregs compared to primary lung tumors and an increase in CD204+ cells [20]. Recent studies by Dykema et al. [34] have integrated single-cell TCRseq/RNAseq data from human NSCLC patients and murine tumors to reveal distinct Treg subclusters within the TME. An “activated” Treg subcluster, defined by the expression of TNFR superfamily genes OX40 and GITR, was highly suppressive and associated with resistance to immune checkpoint inhibitors. In murine models, most tumor-reactive Tregs evolved into a proinflammatory Th1-like phenotype, also prevalent in human tumors responding to anti-PD-1 therapy. These findings highlight tumor-associated Treg heterogeneity and suggest that targeting specific Treg subclusters may improve ICI responses. Furthermore, research by another team has demonstrated that T cell-mediated tumor reactivity manifests in brain metastases, characterized by the enrichment of CD39+ T cells expressing CXCL13, which are potential tumor-reactive T cells (pTRT) [35].

#### 2.2.2. Cancer-Associated Fibroblasts

Cancer-associated fibroblasts (CAFs) represent a pivotal cellular component in the tumor microenvironment of non-small-cell lung cancer, contributing significantly to disease progression and brain metastases. Activated by factors such as TGF-β and exosomes secreted from NSCLC cells, CAFs adopt a pro-tumorigenic role, secreting a myriad of cytokines, chemokines, and growth factors [36,37]. These secretory products modulate the behavior of various immune cells, including CD8+ T cells, regulatory T cells, and macrophages, further contributing to immune suppression and pro-angiogenic processes. A critical mechanism through which CAFs exert immunosuppression is via the release of the chemokine CXCL12, which engages the receptor CXCR4 on cancer cells and leads to T cell exclusion [38,39]. This interaction emphasizes the role of fibroblast activation protein-a (FAP-a) in immune evasion. Several studies underscore the functional diversity within the CAF population. For instance, Hu et al. [40] identified an abundance of CAFs not only in primary tumors but also in brain metastases, while Akanda et al. linked the elevated expression of CAF-related biomarkers such as PDGFR-β and α-SMA to poor prognosis and recurrence in NSCLC brain metastases [41]. On the molecular level, CD10+GPR77+ and CD44+ CAF subsets have been implicated in maintaining tumor stemness. Furthermore, novel regulatory pathways have been proposed. Kim et al. [42] reported that apoptotic NSCLC cells reprogram CAFs through Notch1-WISP-1 signaling, which inhibits the migratory and invasive properties of both cancer cells and CAFs. Zhang et al. [43] demonstrated that CAFs protect NSCLC cells from irradiation-induced DNA damage by promoting DNA repair and cell cycle arrest in the radioresistant S phase. Through these multifaceted interactions and secretory functions, CAFs substantially alter the TME, rendering it more conducive for NSCLC progression and brain metastases. Thus, understanding the specific roles and functional heterogeneity of CAFs is crucial for the development of targeted therapies for NSCLC brain metastases.

#### 2.2.3. Tumor-Associated Macrophages

Tumor-associated macrophages (TAMs), constituting approximately 30% of the immune infiltrate, are prominent players in the TME of NSCLC and its corresponding brain metastases. Exhibiting remarkable plasticity, TAMs can differentiate into distinct phenotypes—M1, which are pro-inflammatory and anti-tumorigenic, and M2, which are immunosuppressive and pro-tumorigenic—based on specific cues within the TME [44]. Notably, M2-polarized TAMs are associated with more aggressive tumor phenotypes, facilitating invasion and metastasis through the secretion of cytokines like IL-6, IL-8, and IL-10. These M2 TAMs also modulate the expression of programmed cell death-ligand 1 (PD-L1) on both tumor cells and infiltrating immune cells, contributing to immune evasion and disease progression in NSCLC patients [44,45]. Recent studies have elucidated the molecular underpinnings of TAM functionality in NSCLC brain metastases. Xiao et al. [24] reported that EGFR-positive and ALK-positive brain metastases are characterized by an immunosuppressive milieu, with decreased CD8+ T cells and increased regulatory T cells (Tregs) or M2 macrophages, respectively. Xu et al. [46] demonstrated that extracellular vesicle (EV)-derived long non-coding RNA LINC00482 modulates the miR-142-3p/TGF-β1 axis, inducing M2 polarization in microglia and fostering a pre-metastatic niche for NSCLC brain metastases. Other research has implicated exosomal miR-155, miR-196a-5p, and miR 942 from M2 TAMs in promoting NSCLC metastasis [47,48]. Moreover, Tiong et al. established that exosomal miR-21 drives lung-to-brain metastases through the DGKB/ERK axis within the TME [49]. Intriguingly, single-cell RNA sequencing identified IL6 as a critical regulator in brain metastatic NSCLC cells, inducing anti-inflammatory microglia via JAK2/STAT3 signaling and promoting colonization, as indicated by Jin et al. [50]. Targeting this IL6/JAK2/STAT3 pathway in activated microglia offers a promising avenue for inhibiting brain metastases in NSCLC. Recent advancements in imaging technologies, like fluorine isotope 19 magnetic resonance imaging (19F MRI), have even enabled the non-invasive tracking of TAMs over time, as demonstrated by Croci et al. [51]. Through these complex interactions and regulatory mechanisms, TAMs are shaping the immunosuppressive landscape of NSCLC brain metastases, offering multiple avenues for targeted therapies.

#### 2.2.4. Astrocytes

Astrocytes are the most abundant mesenchymal cell type within the central nervous system (CNS) and play a pivotal role in maintaining homeostasis. In the context of brain metastases from NSCLC, they form a crucial component of the tumor microenvironment (TME) and exhibit a complex and dual behavior [52]. Initially, astrocytes act defensively against invading cancer cells. They produce plasminogen activator (PA), converting plasminogen into plasmin, activating the pro-apoptotic cytokine Fas ligand, which serves as a defense mechanism against exudative metastatic cells and inhibits the spread of tumor cells along intracerebral capillaries. However, tumor cells develop several convoluted countermeasures against these defensive mechanisms. For instance, they secrete anti-PA serine protease inhibitors such as neuroserpin and serpin B2, thereby resisting Fas-mediated apoptosis. Adler et al. [53] demonstrated that lipocalin-2 (LCN2), primarily secreted by granulocytes, induces the inflammatory activation of astrocytes, subsequently attracting myeloid cells to the brain and promoting neuroinflammation. Interestingly, the study found that the genetic targeting of LCN2 or bone marrow transplantation from LCN2−/− mice attenuated neuroinflammation and inhibited brain metastasis. Astrocytes also interact with tumor cells by assembling connexin 43 gap junctions, mediated by intracellular pro-calmodulin 7 [54]. This interplay induces innate immune responses that paradoxically promote tumor growth and chemoresistance via intracellular STAT1 and NF-kB pathways [55]. Ma et al. [56] discovered that IFN signaling in astrocytes activates the production of C-C motif chemokine ligand 2 (CCL2), recruiting monocytic myeloid cells and thus facilitating brain metastasis, despite IFN’s traditionally considered anti-tumor effects. Ye et al. [57] further contribute by showing that exosomes from NSCLC cells can induce apoptosis in astrocytes and promote the secretion of cytokines that facilitate an inflammatory and immunosuppressive microenvironment, thus setting the stage for the formation of a pre-metastatic niche in lung cancer brain metastases. Recent studies have shown that activated astrocytes can be identified by phosphorylated STAT3 (pSTAT3) expression, instrumental in altering the TME to facilitate brain metastasis [58]. Astrocytes also secrete miRNA-142-3p, inhibiting the transient receptor potential ankyrin-1-mediated activation of fibroblast growth factor 2, offering another avenue for brain metastasis mitigation [59]. Astrocytes exhibit a complex relationship with metastatic NSCLC cells, initially acting as a defense but eventually facilitating tumor growth due to intricate molecular interactions and their relationship with various immune cells [15,52]. These findings underscore the complexity of targeting astrocyte interactions in seeking effective therapies against NSCLC brain metastases.

### 2.3. Molecular Landscape of Brain Metastases

The molecular intricacies of NSCLC brain metastases are complex. NSCLC itself demonstrates an augmented tendency to give rise to brain metastases, especially in the context of ALK fusions and EGFR mutations [60,61]. This propensity remains pronounced even when systemic malignancy is controlled through targeted therapeutics or immunotherapeutic strategies. The inefficacy of early generation ALK inhibitors to sufficiently penetrate the central nervous system further amplifies the risk of CNS-based progression. In a comprehensive genomic study, Huang et al. [62] highlighted the brain metastases-specific increase in ALK fusions, KRAS G12C mutations, and MET amplifications compared to their primary NSCLC counterparts. Nonetheless, the frequency of MET exon14 skipping mutations was significantly reduced. Whole-exome sequencing of lung adenocarcinoma brain metastases further revealed that the prevalence of pathogenic genomic alterations, such as MYC, YAP1, and MMP13 amplifications and CDKN2A/B deletions [63]. In the context of primary squamous cell carcinoma (SCC), the aberrant PI3K pathway is linked to worse survival and increased brain metastasis incidence [64]. Other investigations also detected the aberrations in the PI3K/AKT pathway in both primary and brain metastases tissues, underscoring the pivotal role of this pathway in BM evolution [65]. A wide-angled whole-genome sequencing (WGS) approach revealed a homogenous pattern of heterozygous PTEN loss across all brain metastases in patients with SCC [65,66]. These findings highlight the unique genomic basis of brain metastases and the intricate clonal heterogeneity compared to primary tumors.

Recent studies have significantly advanced our understanding of brain metastases on a molecular basis. A comparative genomic study that investigates the difference between brain metastases and extracranial metastases revealed unique BCL6 and NOTCH2 variants as brain metastases-specific markers [67]. Additionally, a panel of 20 genes, including TP53, SMAD4, SF3B1, NOTCH2, and others, presented as prevalent variants in a significant fraction of patients with brain metastases, suggesting a potential genetic signature for brain metastases susceptibility that requires further validation [62,67]. Using Illumina RNA sequencing, a cohort study involving 91 NSCLC patients (32 developed brain metastases) identified a panel of 22 genes that were distinctly associated with brain metastases, exhibiting no correlation with other metastatic sites. The next-generation sequencing (NGS) of 416 cancer-associated genes from primary lung tumors and paired brain metastases reported over 80% concordance for mutations in EGFR, KRAS, TP53, and ALK, underscoring the parallel evolution of primary tumors and brain metastases [68]. For instance, synchronous brain metastases were found to possess a greater burden of cancer-associated mutations compared to primary tumors, suggesting an accelerated genomic evolution driven by the activation of supplementary oncogenic mechanisms. Conversely, this observation was not mirrored in follow-up studies. Others suggest that protein levels of TYMS, CDK1, CEP55, HJURP, and KIF11 could potentially forecast brain metastases incidence in lung adenocarcinoma patients [69]. Additionally, using single-cell transcriptomic sequencing uncovered substantial disparities in immune landscapes between primary tumors and brain metastases, and a unique metastatic gene signature rich in metabolism and cell adhesion-related genes. Zhang et al. explored gene expression differences between primary lung adenocarcinomas and their corresponding brain metastases using two patient-derived xenograft models. The research highlights CKAP4, SERPINA1, SDC2, and GNG11 as genes that are differentially expressed in these malignancies, proposing their utility as potential biomarkers. These biomarkers could significantly enhance prognostic assessments and inform therapeutic strategies for lung cancer patients with brain metastatic involvement [70]. A variety of gene classes associated with metastasis present modified DNA methylation patterns, correlating with altered gene expression in metastatic samples, including hypermethylation of the CDH1 promoter and subsequent reduction in expression [71]. Karlow et al. [72] revealed that lung cancer brain metastases are characterized by altered DNA methylation patterns. This change is associated with a reduction in EZH2 binding at genes crucial for development, potentially facilitating the emergence of stem-like traits that allow cancer cells to spread and thrive in diverse microenvironments. Specifically, an increase in methylation was identified at a locus overlapping the promoter of the ZNF154 gene in metastatic samples when compared to primary tumors. Higher methylation levels at this promoter correlate with a poorer patient prognosis. Furthermore, the most consistent epigenetic alterations observed as the cancer progresses to metastasis include methylation increases within DNA methylation valleys (DMVs). These valleys are marked by constitutive heterochromatin indicator, H3K9me3, alongside the bivalent chromatin modifications H3K27me3 and H3K4me1. Overall, genetic and epigenetic alterations play a crucial role in the development and characterization of brain metastases from NSCLC, offering potential biomarkers for prognosis and targets for therapeutic intervention.

## 3. Translational Biomarkers in NSCLC Brain Metastases

### 3.1. Tissue-Based Molecular Biomarkers

Recently, several FDA-approved therapies have been developed that specifically target genomic alterations in tyrosine kinase-related genes, including EGFR, ALK, ROS1, MET, RET, KRAS, BRAF, and NTRK1-3 [73]. These therapies, accompanied by their respective companion diagnostics, are now utilized in the treatment of NSCLC. The National Comprehensive Cancer Network (NCCN) guidelines currently endorse the use of next-generation sequencing for the detection of a broad spectrum of potential biomarkers in patients with NSCLC [74]. This includes testing for mutations in ERBB2 and amplifications in MET, which are increasingly recognized as actionable targets for personalized therapy. The landscape of NSCLC has been significantly advanced by biomarker testing, particularly in the challenging field of brain metastases. This complex issue affects an ethnic range, with 10–20% of Caucasians and up to 60% of Asians exhibiting molecular alterations at diagnosis [75]. Moreover, mutations in the EGFR gene, along with fusions in the ALK and RET genes, serve as critical driving factors for the progression of brain metastasis in patients with advanced NSCLC [76]. EGFR mutations, present in around 40% of Asian NSCLC patients, emphasize the necessity for routine testing, especially as a reported discordance rate up to 27% exists between extracranial and intracranial specimens in brain metastases patients. In NSCLC brain metastases, *EGFR* and *ALK/ROS1* inhibitors show promising results in patients harboring these genomic alterations [77]. The third-generation EGFR-TKI osimertinib has shown remarkable intracranial response rates over 80% in metastatic cases, a significant step in treating brain metastasis [78]. The importance of EML4-ALK fusion protein, expressed in 2–9% of lung adenocarcinomas, has led to the recommendation of ALK testing in non-squamous histology if EGFR mutations are not detected. Next-generation ALK inhibitors, including alectinib and lorlatinib, have been associated with considerable intracranial response rates over 65%, showcasing their effectiveness in treating brain metastases [79]. ROS1 gene rearrangements, although not routinely screened, are present in 1–2% of non-squamous NSCLC patients, and their significant intracranial response rates with TKIs should prompt regular ROS1 testing, particularly in brain metastases patients [80]. BRAF mutations, detected in 2–4% of NSCLC patients, have revealed the clinical efficacy of targeted treatments like dabrafenib and trametinib, even in brain metastases, though routine testing is not universally recommended. Additionally, MET amplification emerges as an independent predictor of poor survival in brain metastases [81]. New insights into tissue-based biomarkers for NSCLC brain metastasis have emerged from recent studies. For instance. RAC1, notably enriched in metastatic LUAD tissues, is implicated in promoting brain metastases, with high expression levels indicating a poorer prognosis [82]. The increased expression of activated leukocyte cell adhesion molecule (ALCAM) is significantly correlated with upregulation in NSCLC brain metastases, leading to reduced survival times [83]. Brain-derived neurotrophic factor (BDNF) levels in primary LUAD have been linked to a heightened risk of brain metastasis development, with brain metastases expressing higher BDNF levels than their primary lesion counterparts [84]. Furthermore, other tissue markers, including cholecystokinin A receptor (CCKAR) and WD repeat domain 5 (WDR5), are significantly associated with brain metastasis and have emerged as independent risk factors [85,86].

**Figure 2:**
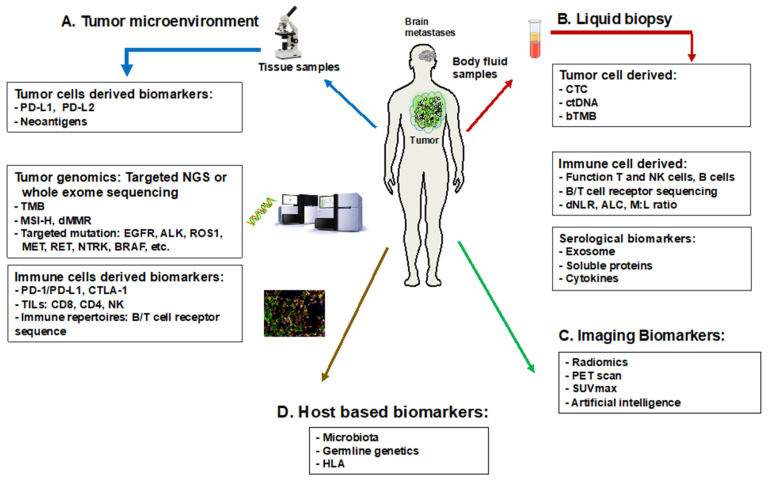
Predictive translational biomarkers for brain metastases of NSCLC. Abbreviations: ALK: anaplastic lymphoma kinase; ALC: absolute lymphocyte count; BRAF: B-Raf proto-oncogene, serine/threonine kinase; ctDNA: circulating tumor DNA; bTMB: blood tumor mutational burden; dMMR: deficient mismatch repair; dNLR: derived neutrophil-to-lymphocyte ratio; CTC: circulating tumor cells; CTLA-4: cytotoxic T-lymphocyte-associated protein 4; EGFR: epidermal growth factor receptor; HLA: human leukocyte antigen; MET: mesenchymal–epithelial transition factor; M:L ratio: monocyte-to-lymphocyte ratio; MSI-H: microsatellite instability—high; NK: natural killer cells; NGS: next-generation sequencing; PD-L1: programmed death-ligand 1; RET: rearranged during transfection; TILs: tumor-infiltrating lymphocytes; TMB: tumor mutational burden; ROS1: c-ros oncogene 1; SUVmax: maximum standardized uptake value.

The advent of PD-1 and PD-L1 immune checkpoint inhibitors has significantly reshaped the treatment landscape for metastatic NSCLC, particularly in managing brain metastases [87]. These therapies, as monotherapies or combined with chemotherapy, have presented substantial intracranial responses. Especially in NSCLC patients with high PD-L1 levels (≥50% for first-line and ≥1% for second-line treatments), significant benefits have been observed [88]. However, PD-L1 expression in NSCLC varies widely, ranging from 50% to 70%, reflecting the complex heterogeneity between tumors. Notably, a strong correlation between PD-L1 expression in primary NSCLC and brain metastases was found, for which the concordant rate ranged from 70 to 90%, whereas CD8+ TILs density was concordant in only about 50% of paired samples [89]. Immune checkpoint inhibitor-based treatment yielded similar intracranial and extracranial response rates. Interestingly, PD-L1-positive brain metastases patients exhibited shorter brain-specific disease-free survival than the PD-L1-negative resected brain metastatic group, and those with at least 1% PD-L1 expression had longer overall survival [90,91]. Factors like sex, age, and brain metastases status have been identified as predictive parameters for treatment responses. However, the predictive role of PD-L1 remains suboptimal due to discordant therapeutic responses, varying survival prognosis, non-standardized techniques, and the dynamic and heterogenous expression of PD-L1 during disease progression [92]. As such, the definitive potential of PD-L1 expression in guiding immune checkpoint inhibitor-based therapy in the brain metastases of NSCLC requires further clarification and standardized methodologies. Given this, the poor prognosis of NSCLC brain metastases, and the numerous approved therapies for metastatic NSCLC, the presence of the associated biomarkers for these approved agents within NSCLC brain metastases warrant further study. Figure 2 summarizes the predictive biomarkers in brain metastases of NSCLC.

### 3.2. Liquid Biopsy Biomarkers: Circulating Tumor Cells and ctDNA

While tissue biopsy remains the gold standard for cancer diagnosis, it comes with certain constraints, including the size of the lesion, tumor heterogeneity, the patient’s ability to undergo the procedure, and the tumor’s accessibility. Liquid biopsy, on the other hand, is gaining interest in the scientific community as a minimally invasive technique that permits repeated sampling with minimal risk. Brain metastases in NSCLC are believed to originate from circulating tumor cells (CTCs), which constitute a significant component of liquid biopsies [93]. As most cancers are of epithelial origin, the most common marker used for CTCs is EpCAM. EpCAM expressions are commonly used to detect CTCs, but due to the EMT activity of NSCLC cells, detecting only EpCAM-positive CTCs probably underestimates the actual total CTC population and misses important biological information of EpCAM-negative CTCs [94]. It was even found that the quantity of EpCAM-negative CTCs was significantly larger than EpCAM-positive CTCs in NSCLC [95]. Using the CellSearch^®^ system, one group found that CTCs were detected in only a few NSCLC patients with brain metastases and even fewer in oligo brain metastases. However, oligo-brain NSCLC metastatic patients with CTCs exhibited a very poor prognosis [96]. Scharpenseel et al. [97] found that by combining EpCAM enrichment with EGFR- and HER3-based enrichment, CTCs can be detected in a large proportion of NSCLC patients, including brain metastatic patients who were previously found to have only very few EpCAM positive CTCs.

Circulating tumor DNA (ctDNA) has emerged as a vital biomarker with profound implications in the diagnosis and prognosis of lung cancer brain metastases. Distinct from circulating tumor cells (CTCs), ctDNA allows for a unique exploration of genomic alterations, encompassing changes in tumor suppressor genes, oncogenes, microsatellite instability, and epigenetic modifications [98]. The accessibility of ctDNA eliminates the need for enrichment of rare cell types, enabling more efficient genotyping and assessment of drug responses, relapse screening, and survival prognosis [99,100]. The origins of ctDNA in brain metastases can be traced to neurons, oligodendrocytes, or astrocytes, exhibiting distinct methylation patterns and marked upregulation in serum samples when compared to other extracranial metastases [101]. Remarkably, ctDNA levels are not solely dictated by tumor volume but are also influenced by genetic factors, including mutations in genes such as EGFR or KRAS in non-small-cell lung cancer [102]. Studies on NSCLC brain metastases patients have demonstrated a 70-90% concordance of EGFR mutation status between tissue and serum samples [103]. Monitoring clonal evolution through ctDNA genotyping post-clinical interventions has further cemented its significance. Its role in therapy is also expanding, where ctDNA assessment post-surgery or chemotherapy can stratify patients and identify high-risk candidates for aggressive treatment, even targeting minimal residual disease, thus augmenting cure possibilities.

A deeper understanding of ctDNA also illuminates the complex nature of the metastatic cascade, discerning between monoclonal and polyclonal seeding. Recent research has proposed plasma ctDNA circulating tumor fraction (TF cut-off 10) as an independent prognostic biomarker across advanced cancers [104]. Although blood-based liquid biopsy offers non-invasive biomarker screening, cerebrospinal fluid (CSF) is an alternative biopsy type to evaluate the genetic landscape of intracranial tumors, particularly in cases of brain metastases. Li et al. [105] found that a higher number of unique copy number variations was detected in CSF-ctDNA than in plasma, and ctDNA positivity of CSF samples at baseline was associated with poor-outcome NSCLC brain metastases. Patients who achieved a reduction of 50% or greater in CSF ctDNA levels following an 8-week treatment regimen experienced notably extended intracranial progression-free survival (PFS) compared to those with reductions of less than 50%. Studies by Wu et al. [106] have underscored CSF ctDNA’s superior ability over plasma ctDNA at representing the mutational landscape of brain metastases, detecting all brain metastases mutations in 83.33% of patients. Mutant allele frequency (MAF) in CSF ctDNA has further shown a strong correlation with brain metastases tumor size. The utilization of targeted next-generation sequencing analysis of ctDNA and exosomal RNA in CSF and plasma has revealed substantial overlaps in variants between primary lung tumors and brain metastases. Specific in-frame deletions and missense mutations were identified, and correlations with brain metastases were unveiled. Detection frequencies of up to 90% in CSF and around 60% in blood were noted [107,108]. Mutations in genes like KEAP1, NRF2, P300, ALK, and RET were discovered in brain metastases, contributing insights into cellular survival during circulation [109]. ctDNA in the context of lung cancer brain metastases presents a multifaceted and promising field for enhanced diagnosis, prognosis, and tailored therapeutic strategies. In addition, the introduction of CSF as a new form of liquid biopsy has enabled higher detection rates of specific EGFR mutations in NSCLC brain metastases patients, contributing to remarkable response rates with EGFR-TKI treatment [110]. However, certain aspects of its mechanism and characteristics necessitate further exploration, underscoring the ongoing importance of research in this domain.

### 3.3. Exosomes and Non-Code RNA

Exosomes, extracellular vesicles ranging between 50 and 200 nm in diameter, are integral components of intercellular communication, immune response, and various pathological states, including inflammation, neurodegeneration, and particularly cancer [111]. Within the context of lung cancer’s propensity to metastasize to the brain, a notable increase in exosomal release has been observed [112]. These vesicles harbor critical components such as DNA, RNA, microRNA, proteins, and metabolites, all of which play a pivotal role in cancer proliferation and metastatic progression. Consequently, they hold significant promise for both diagnostic applications and targeted therapy [113]. In the intricate landscape of lung cancer, exosomes actively shape the tumor microenvironment, fostering tumor growth and facilitating distant metastasis by promoting the formation of pre-metastatic niche in brain metastases and induce astrocyte apoptosis by affecting cytokine secretion, protein expression, and energy metabolism [57,114]. The presence of unique surface proteins in exosomes provides valuable clues to their cellular origin, thus assisting in pinpointing preferred metastatic destinations [115]. Lung cancer-derived exosomes have been observed to compromise the integrity of the blood–brain barrier, instigating microglia activation [116]. This underscores their significance beyond mere diagnostic or prognostic markers. Emerging isolation techniques have broadened the scope of employing exosomes in diagnostics, therapy, and continuous treatment monitoring. By isolating and characterizing these vesicles, we can unravel complex challenges pertaining to drug efficiency or resistance in brain metastases.

Profiling the proteins within exosomes allows for precise differentiation between tumorous and normal cells, with multi-panel profiling demonstrating effectiveness in predicting tumor origin [117]. Exosomes enriched with specific proteins can condition the brain microenvironment to facilitate cancer cell proliferation, as evidenced by the elevated colonization and invasiveness observed in brain tissues treated with exosomes derived from brain metastatic cells [118]. Exosome integrins, known for their interaction with extracellular matrix proteins and regulation of cell survival and metastasis, present in varying amounts in different metastatic sites. Recent studies utilizing brain-tropic cell exosomes have revealed an upregulation of integrins, including ITGβ3, in brain metastases NSCLC models [115,119]. Our research group has also successfully applied the high-affinity peptide ligand LXY30 to target α3β1 integrin in NSCLC’s exosomes [120].

Non-coding RNAs (ncRNAs), including microRNAs (miRNAs) and long non-coding RNAs (lncRNAs), are vital in the biology of lung cancer brain metastasis. MiRNA-378, for example, promotes brain metastases in NSCLC by modulating the genes involved in angiogenesis and extracellular matrix invasion [121], while the downregulation of miRNA-145 leads to brain metastases in lung adenocarcinoma [122]. PTEN regulation through microRNAs, downregulated in cancer cells but restored when leaving the brain microenvironment, demonstrates microRNAs’ critical role in brain metastases [123]. lncMMP-2 is highly expressed in TGF-β-mediated exosomes. It increases blood–brain barrier permeability via the miRNA-1207-5p/EPB41L5 axis and leads to NSCLC brain metastases [124]. MiR-335-5p and miR-34b-3p were identified as unique to NSCLC brain metastasis, with others such as miR-330-3p having a diagnostic potential [125,126]. Comparative studies identified specific miRNAs upregulated and downregulated in brain metastases compared to primary NSCLC tumors, highlighting the role of miRNAs in controlling tumor cell proliferation, migration, and invasion [127]. Notable findings include miR-596-3p’s role in inhibiting brain metastases by modulating YAP1 and IL-8 and the significant downregulation of microRNA-375 in patients with brain metastases [128,129]. This intricate network of ncRNAs reveals promising diagnostic possibilities and emphasizes the need for continued exploration to enhance early detection and develop tailored therapies against metastatic lung cancer.

### 3.4. Imaging Biomarkers

The emerging evidence indicates that NSCLC with different features exhibits variability in imaging characteristics of the primary tumor and metastatic locations. The procurement of high-quality medical imaging through computed tomography and magnetic resonance imaging (MRI) is essential in the diagnostic process and treatment stratification for brain metastases [130]. Radiomics, a cutting-edge approach that utilizes advanced computational techniques to extract high-throughput quantitative features from medical images, has garnered considerable attention for its utility in characterizing various types of brain metastases, especially those originating from NSCLC [131]. Notably, work by Kneipp et al. [132] established that machine learning algorithms could be employed in conjunction with radiomic features to predict the histological origin of brain metastases with significant accuracy. Their study validated that quantitative metrics gleaned from routine magnetic resonance imaging (MRI) scans, when processed by machine learning classifiers, could offer a high level of discriminatory accuracy in pinpointing the tumor origin of these metastases. In a parallel vein, Zhou et al. [133] found that when radiomic features are amalgamated with machine learning methodologies, there is a powerful discriminatory capacity to distinguish between primary lung lesions and metastatic lung lesions in the brain. Intriguingly, emerging datasets also indicate that NSCLCs harboring different targetable oncogenic driver mutations (e.g., ALK) present unique imaging features, both in the primary tumor and in metastatic tumors [134,135]. Specifically, the utility of contrast-enhanced T1-weighted (CET1W) images in predicting EGFR mutation status in small-sized brain metastases (<10 mm) from lung cancer has been highlighted [136]. Moreover, our group has made pivotal contributions in elucidating the synergistic potential of combining radiomic imaging biomarkers with conventional molecular markers. Our team found that the LXY30-biotin/streptavidin-Cy5.5 complex shows preferential uptake in intracranial xenografts of α3β1 integrin-expressing lung adenocarcinomas while sparing the surrounding normal tissues, suggesting its viability as a robust and specific biomarker for lung–brain metastases [120]. To transition these groundbreaking findings from the research phase to clinical application, it is paramount that the diagnostic and prognostic accuracy of these imaging biomarkers be substantiated through rigorous validation in multiple clinical settings to ensure both reproducibility and generalizability.

### 3.5. Immune Cells and Serological Biomarkers

Peripheral immune biomarkers in NSCLC present a multifaceted landscape, comprising many components that undergo alterations during natural tumor progression and therapeutic interventions. Our prior investigations have highlighted that anti-cancer agents, including small molecule tyrosine kinase inhibitors and immune checkpoint inhibitors (ICIs), exert modulatory effects on the immune milieu and peripheral blood immune cells of advanced NSCLC patients [137]. For instance, the emergence of post-treatment lymphopenia was discernibly linked with suboptimal clinical outcomes in this patient cohort [138]. Diving deeper into the prognostic potential of immune parameters, several groups uncovered that the neutrophil–lymphocyte ratio (NLR) and serum albumin levels served as independent harbingers of enhanced survival in NSCLC patients with brain metastasis [139]. When NLR and albumin metrics were integrated to conceive the modified systemic inflammation score (mSIS), it outperformed the discriminative capability of the AJCC’s seventh T + N staging system [140]. In parallel, another research contingent identified the prognostic relevance of the prognostic nutritional index (PNI) and systemic immune inflammation index (SII) for NSCLC patients manifesting brain metastases [141]. Exploiting multicolor spectrum flow cytometry, our team recently pinpointed that ICIs notably influenced the prevalence of blood cytotoxic CD16+CD56dim, CD16+CD56- natural killer cells, and CD14+HLDRhigh monocytes [142]. These subsets bore significant prognostic associations in advanced NSCLC, indicating their prospective utility as monitoring tools in NSCLC brain metastases therapeutic paradigms. On the serological front, proteomics emerges as a promising avenue for the early detection and surveillance of NSCLC brain metastases patients [143]. A few previous studies have drawn correlations between pretreatment serum carcinoembryonic antigen (CEA) levels and brain metastasis in advanced NSCLC [144]. In a recent endeavor, Wei et al. [145] spotlighted the upregulated profiles of cathepsin F (CTSF) and fibulin-1 (FBLN1) in serum and tissue specimens of NSCLC brain metastasis patients. Intriguingly, serum alterations in CTSF mirrored the therapeutic trajectories of these patients, even preceding detectable changes in magnetic resonance imaging. Elevated tissue levels of CTSF correlated with diminished progression-free survival (PFS), establishing its stature as an independent prognostic marker. Concurrently, Chen et al. noted that elevated serum S100B levels were directly proportional to the burden of brain metastases in NSCLC, portending an adverse prognosis [146]. Such serological markers undeniably warrant exhaustive validation in subsequent studies.

### 3.6. Microbiota

The complex interplay between the microbiota and carcinogenesis is increasingly evident, particularly in NSCLC pathogenesis. The lung and gut microbiota have been intricately associated with NSCLC, yet their distinct contributions, especially regarding brain metastases, remain elucidated [147]. Lu et al. [148] reported a notable enrichment of *Pseudomonas aeruginosa* in the sputum of patients with brain metastases, a bacterium classically linked to wound infections. Remarkably, this species was predominantly observed in the sputum of brain metastases patients compared to other forms of NSCLC or distant metastases. Notably, the activation of toll-like receptor 4 by Gram-negative bacteria enhances metastasis in non-small-cell lung cancer through the phosphorylation of mitogen-activated protein kinases [149]. In a parallel investigation, Huang et al. [150] conducted a sequencing analysis of bronchoalveolar lavage fluid in 40 NSCLC cases. Their findings indicated a marked reduction in Streptococcus content in metastatic lung adenocarcinoma compared to its non-metastatic counterpart. Conversely, in lung squamous cell carcinoma, the proportions of Veillonella and Rothia were considerably elevated in metastatic cases compared to non-metastatic ones. The gut–brain axis (GBA) is a critical communication pathway where the gut microbiota can influence brain disorders. Within this framework, metabolites play a pivotal role as mediators, traversing into the bloodstream to exert their functional impact. Significantly, there is evidence to suggest that the activation of microglia has a positive correlation with brain tumor progression [151]. Li and colleagues further illuminated the intricate relationship between gut microbiota and NSCLC brain metastasis [152]. They highlighted the role of a specific bacterium, intestinimonas, and its associated plasma metabolite, spermine, in augmenting brain metastasis in NSCLC. This research elucidated a novel intestinimonas–spermine model, which offers potential as a biomarker for evaluating brain metastasis risk in NSCLC and sheds light on the mechanistic pathway involving STAT3 signaling. Notably, this signaling pathway accentuates the polarization of microglia towards a pro-tumor M2 phenotype, subsequently propelling brain tumor growth. While an association between microbiota dysbiosis and NSCLC has been established, precise mechanisms warrant further investigation, particularly in brain metastasis.

## 4. Conclusions and Future Therapeutic Implications

The treatment of brain metastases in NSCLC presents a complex clinical challenge. Guided by comprehensive guidelines, decisions often require a multidisciplinary approach, incorporating key factors such as the patient’s Karnofsky performance status and the primary cancer’s origin. Local interventions like stereotactic radiosurgery (SRS) and surgical resection remain valuable options, particularly for limited metastatic burden within the brain [153]. Advanced surgical techniques, such as cortical mapping, are increasingly employed to minimize perioperative morbidity. The advent of molecular diagnostics and targeted therapies has profoundly impacted brain metastases management in NSCLC. Broad molecular profiling is essential, especially for patients with metastatic non-squamous NSCLC. TKIs, for instance, have shown significant efficacy in controlling CNS metastases, frequently obviating the immediate need for local therapies like surgery or whole-brain radiation therapy. Table 1 outlines recent clinical studies on NSCLC brain metastasis treatments. In asymptomatic patients with limited brain metastases burden, first-line TKIs can often defer local treatment and mitigate the risk of CNS progression. Liquid biopsy offers a non-invasive, timely means of identifying actionable mutations and evaluating TKI resistance mechanisms. While immune checkpoint inhibitors have ushered in a new era in NSCLC treatment, their role in managing brain metastases remains an area of ongoing research, with current evidence indicating modest intracranial effectiveness [154,155]. A summary of recent clinical trials evaluating NSCLC brain metastases treatment can be found in Table 2. Future investigations should prioritize phase II and III trials that explicitly assess intracranial efficacy as a secondary endpoint. Concurrently, there is an imperative need to develop companion diagnostic and prognostic biomarkers for more nuanced risk stratification and treatment planning. In the era of precision therapy for NSCLC brain metastases, management mandates a synergistic collaboration among surgeons, medical oncologists, neurologists, radiologists, and pathologists, each bringing unique insights to revolutionize treatment approaches. There is an urgent need to optimize biomarker-guided treatment selection and precisely define treatment durations to significantly improve survival outcomes in NSCLC brain metastases. Future research should focus on developing innovative biomarkers, understanding basic molecular mechanisms, and enhancing diagnostic approaches.

## Figures and Tables

**Table 1:** Summary of reported clinical efficacy of drugs in NSCLC brain metastases.

Clinical Trials Identifier	Drug Class	Drug Name	Phase of Clinical Trial	Number of Patients	Biomarker	Disease	Median OS	PFS	HR	ORR/DCR	Ref
NCT04211090	PD-1/L1 inhibitor	Camrelizumab, Pemetrexed, Carboplatin	2	45	Driven Gene-negative	NSCLC Brain Metastases	21 months	7.6 months	NA	46.7%/34%	[156]
NCT03769103	TKI	Osimertinib and Stereotactic Radiosurgery (SRS)	2	66	EGFR Mutation	EGFR-Mutated NSCLC With Brain Metastases	25.2	7.1 months	NA	40.5%/NA	[157]
NCT04345146	Antiangiogenic agents	Fractionated stereotactic radiation therapy (FSRT) combined with Bevacizumab	2	108	None	Non-Squamous Non-Small-Cell Lung Cancer with Brain Metastases	NA	15 months	NA	NA/NA	[158]
NCT05326425	TKI	Lazertinib (YH25448)	2	40	EGFR Mutation	Asymptomatic or Mild Symptomatic Brain Metastases NSCLC	Not reached	Not reached	NA	57.9%/97.4%	[159]
NCT03526900	PD-1/L1 inhibitor	Atezolizumab in Combination with Carboplatin Plus Pemetrexed	2	40	EGFR mutation and ALK fusion negative	NSCLC with Asymptomatic Brain Metastasis	13.6	8.9 months	0.99; 95% CI 0.35 to 2.12	NA/NA	[160]
NCT05104281	Antiangiogenic agents	Osimertinib oral and bevazizumab intravenously	3	52	EGFR Mutation	NSCLC Brain Metastases	Not reached	22 months	NA	82.7%/96.2%	[161]
NCT04507217	PD-1/L1 inhibitor	Tislelizumab, Carboplatin, Pemetrexed	2	36	None	Non-Squamous Non-Small-Cell Lung Cancer Brain Metastatic	Not reached	Not reached	NA	56.7%/96.7%	[162]
NCT03653546	TKI	AZD3759, Erlotinib, Gefitinib	2, 3	439	EGFR Mutation	NSCLC Brain Metastases	NA	9.6 months	NA	68.60%	[163]
NCT02864992	MET tyrosine kinase inhibitor	Tepotinib	2	313	MET exon 14 skipping mutation	Patients with advanced or metastatic NSCLC with a confirmed MET exon 14 skipping mutation	19.6 months	11.2 months	NA	51.4%/NA	[164]
NCT04768491	TKI	Dacomitinib	Ambispective cohort study in real world	32	EGFR Mutation	Advanced NSCLC with EGFR Mutations and Brain Metastasis	NA	Not reached	NA	87.5%/100%	[165]
NCT04339829	TKI	Dacomitinib	2	30	EGFR Mutation	EGFR Mutation-Positive NSCLC	NA	17.5 months	NA	96.7%	[166]
NCT03046992	TKI	Lazertinib	Phase 1/2	78	EGFR Mutation	EGFR Mutation-Positive NSCLC	Not reached	11.1 months	NA	55.30%/NA	[167]
NCT04248829	TKI	Lazertinib	3	393	EGFR Mutation	EGFR Mutation-Positive NSCLC	Not reached	20.6 months	0.45; 95% CI, 0.34 to 0.58	76%/NA	[168]
CA209966	PD-1/L1 inhibitor	Nivolumab	NA	409	None	Advanced Non-Squamous Non-Small-Cell Lung Cancer	8.6 months	3 months	NA	17%/39%	[169]

Abbreviations: ALK: anaplastic lymphoma kinase; EGFR: epidermal growth factor receptor; NSCLC: non-small-cell lung cancer; NA, not available; MET: mesenchymal–epithelial transition factor; PD-1, Programmed cell death protein 1; PD-L1: Programmed death-ligand 1; TKIs, tyrosine kinase inhibitor.

**Table 2:** Summary of ongoing clinical trials for lung cancer (NSCLC) brain metastases.

Clinical Trials Identifier	Status	Phase of Clinical Trial	Estimated Enrollment	First Posted	Estimated Completion Date	Intervention/Treatment	Condition or Disease	Biomarker
NCT04889066	Not yet recruiting	2	46	2021.5	2025.1	Durvalumab and radiotherapy	NSCLC Brain Metastases	PD-L1 Expression ≥ 1%
NCT04978753	Recruiting	2	54	2021.7	2023.12	Anlotinib	NSCLC Brain Metastases	EGFR Mutation
NCT05812534	Not yet recruiting	2	36	2023.4	2025.6	Cadonilimab combined with bevacizumab and chemotherapy	NSCLC Brain Metastases	EGFR and ALK negative
NCT05012254	Recruiting	2	71	2021.8	2026.12	Platinum-based chemotherapy plus nivolumab and ipilimumab	NSCLC Brain Metastases	N/A
NCT05746481	Not yet recruiting	2	35	2023.2	2026.12	Tiragolumab combined with carboplatin, pemetrexed, and atezolizumab	Non-Squamous Non-Small-Cell Lung Cancer (NSCLC) and Untreated Brain Metastases	N/A
NCT04824079	Recruiting	2	30	2022.4	2024.7	Keynatinib	EGFR-Mutated NSCLC With Brain Metastases	EGFR Mutation
NCT05800223	Not yet recruiting	3	300	2023.4	2028.12	Armatinib combined with stereotactic body radiotherapy (SRT)	EGFR-Mutated NSCLC With Brain Metastases	EGFR Mutation
NCT04967417	Recruiting	2	50	2021.7	2026.2	Pemetrexed, carboplatin, pembrolizumab	NSCLC Patients with Asymptomatic Brain Metastases	N/A
NCT05840770	Not yet recruiting	2	34	2023.5	2029.1	Cemiplimab	NSCLC Brain Metastases	PD-L1 ≥ 50%
NCT03497767	Recruiting	2	80	2018.4	2024.3	Osimertinib and stereotactic radiosurgery (SRS)	NSCLC Brain Metastases	EGFR Mutation
NCT04905550	Recruiting	2	50	2021.5	2024.8	Craniocerebral radiotherapy combined with Almonertinib	NSCLC Brain Metastases	EGFR Mutation
NCT05522660	Recruiting	3	190	2022.8	2026.12	Immune checkpoint inhibitor-combined stereotactic radiosurgery	NSCLC Brain Metastases	N/A
NCT05477615	Not yet recruiting	2	28	2022.7	2025.6	Lazertinib, pemetrexed, carboplatin	NSCLC Brain Metastases	EGFR Mutation
NCT05567055	Not yet recruiting	2	35	2022.10	2028.5	Capmatinib	NSCLC with Asymptomatic Brain Metastases	MET amplification or METΔex14 detected on cfDNA
NCT04768075	Not yet recruiting	3	200	2021.2	2024.4	Camrelizumab combined with SRT/WBRT and chemotherapy	NSCLC Brain Metastases	Driven Gene-negative
NCT05236946	Recruiting	3	190	2022.2	2026.7	TKI combined with SRS/WBRT	NSCLC with Asymptomatic Brain Metastases	EGFR/ALK Mutation
NCT05807893	Not yet recruiting	2, 3	30	2023.4	2025.3	Serplulimab combined with bevacizumab and first-line chemotherapy	NSCLC Brain Metastases	Negative Driver Gene
NCT04233021	Active, not recruiting	2	69	2020.1	2024.2	Osimertinib	NSCLC Brain Metastases	EGFR Activating Mutation
NCT05180422	Recruiting	1, 2	43	2022.1	2028.6	AMG 510 plus MVASI	NSCLC Brain Metastases	KRAS p.G12C Mutant
NCT04964960	Recruiting	2	45	2021.7	2033.5	Pembrolizumab and Chemotherapy	NSCLC with Asymptomatic Brain Metastatic	Driven Gene-negative
NCT04291092	Recruiting	2	63	2020.3	2023.6	Camrelizumab combined with chemotherapy and local treatment of brain metastases (WBRT, r-knife, SRS, etc.)	NSCLC Brain Metastases	Driven Gene-negative
NCT05146219	Recruiting	2	60	2021.12	2024.12	TY-9591 tablets	NSCLC Brain Metastases	EGFR Activating Mutation
NCT05317858	Recruiting	3	20	2022.8	2024.12	Pembrolizumab	NSCLC Brain Metastases	Driven Gene-negative
NCT04829019	Recruiting	2	88	2021.4	2025.10	Osimertinib and whole-brain irradiation	NSCLC Brain Metastases	EGFR Mutation
NCT04789668	Active, not recruiting	1, 2	10	2021.3	2025.1	Bintrafusp alfa and pimasertib	NSCLC Brain Metastases	N/A
NCT06020066	Recruiting	2	202	2023.8	2029.8	EGFR-TK inhibitor or SRS	NSCLC Brain Metastases	EGFR L858R, EGFR exon 19 deletion
NCT05999357	Not yet recruiting	2	42	2023.8	2028.8	JDQ443	NSCLC Brain Metastases	KRAS G12C
NCT05865990	Not yet recruiting	2	60	2023.5	2026.10	Patritumab deruxtecan (HER3-DXd),	NSCLC Brain Metastases	HER2-Positive
NCT05948813	Recruiting	2	420	2023.7	2027.12	TY-9591	EGFR-Mutated Non-Small-Cell Lung Cancer with Brain Metastases	EGFR-Mutated
NCT06047379	Not yet recruiting	1, 2	134	2023.9	2026.8	NEO212 pembrolizumab or nivolumab	NSCLC expressing PD-L1, with no EGFR or ALK genomic tumor aberrations	PD-L1, no EGFR or ALK genomic tumor aberrations

Abbreviations: ALK: anaplastic lymphoma kinase; EGFR: epidermal growth factor receptor; HER2, human epidermal growth factor receptor 2; KRAS, Kirsten rat sarcoma viral oncogene homologue; NSCLC: non-small-cell lung cancer; N/A, not available; MET: mesenchymal–epithelial transition factor; PD-1, Programmed cell death protein 1; PD-L1: Programmed death-ligand 1; TKIs, tyrosine kinase inhibitor.

## Data Availability

Not applicable.
